# Supplementary material for: Real-World Data of a Group-Based Formula Low Energy Diet Programme in Achieving Type 2 Diabetes Remission and Weight Loss in an Ethnically Diverse Population in the UK: A Service Evaluation
Source: Nutrients. 2022 Jul 30;14(15):3146. doi: 10.3390/nu14153146 (PMC9370492; doi:10.3390/nu14153146)
Supplement: Supplementary file 1 [file nutrients-14-03146-s001.zip › nutrients-1772934-supplementary.pdf]

Supplementary Material

LED intervention overview

Participants were supported in a group setting by the trained HDC MDT. The majority of the sessions were conducted in a group face-to-face setting, however, during the COVID-19 pandemic and subsequent lockdowns, a number of sessions were run via video consultation. Participants all followed a structured LED programme, with groups transitioning from total dietary replacement (TDR), food reintroduction (FR) and weight maintenance (WM) at the same time. The majority of the group sessions were run by one to two DSDs with support from a DSN and Diabetes Psychologist if required – for example, to help collect measurements at baseline, 12 weeks (end of TDR), and 22 weeks (end of food reintroduction) and 12 months (end of weight maintenance).

Group session content was adapted to the diverse community of Hackney. This included portion guidance for Caribbean and African food preferences and signposting to local community health initiatives, including cooking sessions. Participants had the option of attending free cooking sessions provided by a Hackney based charity (Made in Hackney), recipes and resources were centred on Caribbean, African and Southeast Asian cuisine and adapted to ensure healthy eating principles were practiced e.g. lean protein sources, high fibre and unsaturated fat ingredient choices. Carbs and Cals World Food books were provided to participants for reference during group sessions. This resource helped facilitate carbohydrate, calorie and portion control education as well as explore the potential impact of culturally specific foods and cuisine on blood glucose levels. Additionally, participants were encouraged to reflect and share difficulties, successes, and goals during each group session.

Data collection

Participant measurements were completed either before or after each group session by a trained member of the MDT. Measurements were taken at baseline, end of TDR (3 months), FR (5 months) and WM (12 months) the following were collected. All measurements were taken by a HDC Counterweight-Plus trained staff (please see supplementary material) except phlebotomy blood tests.

Due to the COVID-19 pandemic, group 2, 3, 4 were asked, at various stages, to self-report bodyweight, blood glucose levels and blood pressure and report to the MDT. Participants were sent blood glucose meters and testing strips and provided with instructions on self-monitoring blood pressure. Participants were encouraged to conduct fasting blood glucose levels and report these results to the dietitian. If a participant was unable to attend a group session or did not attend (DNA), the MDT tried to arrange a one-to-one appointment (remote or face-to-face) and collect associated measurements accordingly. A participant was defined as a completer if they attended the final session (20).

Exclusion criteria

- Pregnant or planning to become pregnant within the next 6 months
- Breastfeeding
- Had recent weight loss of greater than 5% body weight or is currently on a weight management programme.
- Had or is awaiting bariatric surgery (unless willing to come off waiting list).
- Active cancer
- Heart attack or stroke in last 6 months
- Severe heart failure (defined as New York Heart Association grade 3 or 4)
- Severe renal impairment (most recent eGFR less than 30mls/min/1.73m2)
- Active liver disease (not including Non-Alcoholic Liver Disease)
- Active substance use disorder
- Active eating disorder. Including, binge eating disorder as identified by a score of 27 or greater via the Binge Eating Scale (BES)
- Severe depression as identified by a score of 16 or greater via a Patient Health Questionnaire (PHQ-9)
- Porphyria
- Known proliferative retinopathy that has not been treated.

- Promotion of the programme for groups 1-3 was focused on those diagnosed with T2D for less than 6 years. For group 4, referrals for people diagnosed over 6 years were encouraged.

Methodology for outcomes

- Body Mass Index (BMI) – Height taken via stadiometer at baseline and BMI subsequently calculated throughout programme
- Bodyweight (kg) was measured using Tanita digital bioimpedance scale to the nearest 0.1 kg. Participants were asked to remove any heavy clothing and items.
- Body fat % - Measured via Tanita digital bioimpedance scale
- Blood glucose levels – Measured via random blood glucose levels at the time of appointment. Participants were not instructed to fast beforehand.
- Blood pressure – Measured via digital sphygmomanometer (OMRON). Participants were asked to sit and not talk for 1 minute before taking. The average of three measurements was used.
- HbA1c, total cholesterol (TC), low-density lipoproteins cholesterol (LDL-C), high density lipoprotein cholesterol (HDL-C) and triglycerides – Participants were instructed to have these tests done on the day of the appointment at their local GP practice or the Homerton Hospital Phlebotomy Department
- Rosenberg self-esteem scale – 10-item self-administered questionnaire that measures global self-esteem by measuring positive and negative feelings about the self (Rosenberg, 1965).
- Diabetes Distress Scale (DDS) – Diabetes distress relates to the emotional implications of living with diabetes, including the impact of daily self-management and fear of long-term complications[1].
- Patient Health Questionnaire (PHQ9) – The PHQ-9 is a self-administered valid instrument for screening, provisionally diagnosing, monitoring and measuring depression severity[2]. It was used to objectively determine eligibility and monitor changes and treatment effect throughout the programme.
- Generalised Anxiety Disorder (GAD-7) – The GAD-7 is as a self-administered validated means of measuring the severity of generalised anxiety disorder[3].
- Work and Social Adjustment Scale (WAS) – A self-administered validated scale measuring an individual's perception of work and social functioning and impairment in relation to a specific problem, in this case, T2D[4]. Due to the negative impact of T2D on emotional and psychological wellbeing, the ability of those living with T2D to perform routine social activities may be impacted.
- Binge Eating Scale (BES) – A 16-item self-administered questionnaire to identify behavioural, emotional, and cognitive characteristics of binge eating[5] and programme eligibility. It is recommended individuals with no pre-existing binge eating can safely proceed onto a LED, but risk should be monitored during and on completion[6].

Table S1: Referral data

Characteristics	Participants
Men, n (%)	97 (44.9)
Women, n (%)	119 (55.1)
Age, years (SD)	52.5 (11.0)
Ethnicity, n (%)	
African (incl Somali)	41 (19.0%)
White British	36 (16.7%)
Black British	35 (16.2%)
Any other mixed	30 (13.9%)
Caribbean	29 (13.4%)
Bangladeshi (incl British)	8 (3.7%)
Indian (incl. British)	7 (3.2%)
Other Asian	7 (3.2%)
Other Black	6 (2.8%)
Pakistani (incl British)	5 (2.3%)
Turkish	5 (2.3%)
Not recorded / refused	3 (1.4%)
Irish	2 (0.9%)
Orthodox Jew	1 (0.5%)
Kurdish	1 (0.5%)
BMI, kg/m^2 (SD)	35.7 (6.5)
Duration of diabetes, years (SD)	3.7 (5.2)
HbA1c, mmol/mol (SD)	66.2 (21.1)
Total cholesterol, mmol/L (SD)	4.7 (1.3)
Psychological wellbeing and binge eating scores (SD)	
PHQ9	7.7 (6.4)
GAD7	5.7 (6.0)
WAS	7.5 (7.7)
BES	11.6 (8.9)

Data are in n (%), mean (SD). %, percentage; n, number; BMI, body mass index, HbA1c, glycated haemoglobin; kg/m^2, kilograms per metre squared. kg, kilograms. PHQ9 = Patient Health Questionnaire. GAD7 = Generalised Anxiety Disorder Questionnaire. WAS = Work and Social Adjustment Scale. BES = Binge Eating Scale; mmol/L, millimoles per litre

Table S2: Remission rates within ethnicity groups

	No remission	Remission
**Black British**		
Number	3	4
% within ethnicity	42.9%	57.1%
% within remission	25.0%	17.4%
% of total	8.6%	11.4%
**White British**		
Number	1	6
% within ethnicity	14.3%	85.7%
% within remission	8.3%	26.1%
% of total	2.9%	17.1%
**Caribbean**		
Number	5	1
% within ethnicity	83.3%	16.7%
% within remission	41.7%	4.3%
% of total	14.3%	2.9%
**African (incl. Somali)**		
Number	0	4
% within ethnicity	0.0%	100.0%
% within remission	0.0%	11.4%
% of total	0.0%	11.4%
**Other African**		
Number	1	2
% within ethnicity	33.3%	66.7%
% within remission	8.3%	8.7%
% of total	2.9%	5.7%
**Pakistani (incl. British)**		
Number	1	1
% within ethnicity	50.0%	50.0%
% within remission	8.3%	4.3%
% of total	2.9%	2.9%
**Bangladeshi (incl. British)**		
Number	1	1
% within ethnicity	50.0%	50.0%
% within remission	8.3%	4.3%
% of total	2.9%	2.9%
**Any other mixed**		
Number	0	2
% within ethnicity	0.0%	100.0%
% within remission	0.0%	8.7%
% of total	0.0%	5.7%
**Irish**		
Number	0	2
% within ethnicity	0.0%	100.0%
% within remission	0.0%	8.7%
% of total	0.0%	5.7%

Figure S1: Waterfall Plot showing distribution of weight change of all participants at 12 months

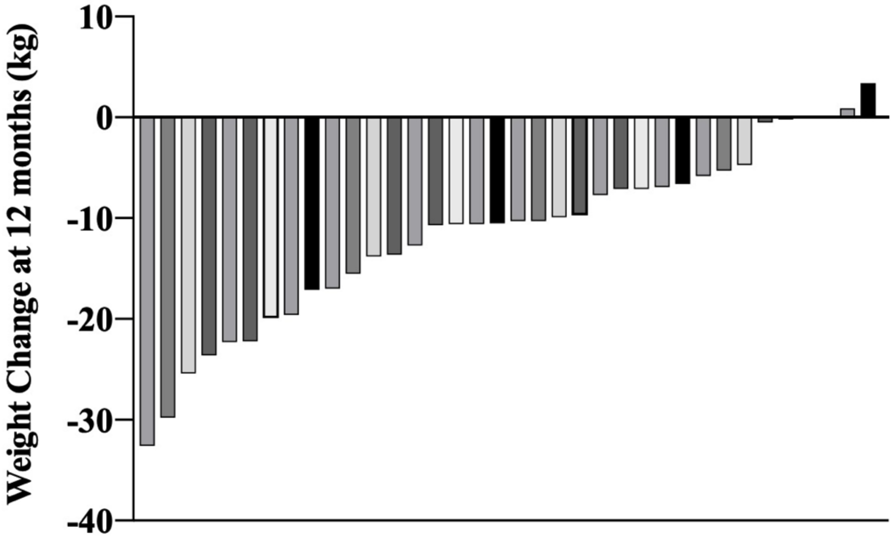

References

1. Polonsky, W.H.; Fisher, L.; Earles, J.; Dudl, R.J.; Lees, J.; Mullan, J.; Jackson, R.A. Assessing Psychosocial Distress in Diabetes: Development of the Diabetes Distress Scale. *Diabetes Care* **2005**, *28*, 626–631, doi:10.2337/DIACARE.28.3.626.

2. Kroenke, K.; Spitzer, R.L.; Williams, J.B.W. The PHQ-9: Validity of a Brief Depression Severity Measure. *Journal of General Internal Medicine* **2001**, *16*, 606, doi:10.1046/J.1525-1497.2001.016009606.X.

3. Spitzer, R.L.; Kroenke, K.; Williams, J.B.W.; Löwe, B. A Brief Measure for Assessing Generalized Anxiety Disorder: The GAD-7. *Arch Intern Med* **2006**, *166*, 1092–1097, doi:10.1001/ARCHINTE.166.10.1092.

4. Mundt, J.C.; Marks, I.M.; Shear, M.K.; Greist, J.H. The Work and Social Adjustment Scale: A Simple Measure of Impairment in Functioning. *The British Journal of Psychiatry* **2002**, *180*, 461–464, doi:10.1192/BJP.180.5.461.

5. Gormally, J.; Black, S.; Daston, S.; Rardin, D. The Assessment of Binge Eating Severity among Obese Persons. *Addictive Behaviors* **1982**, *7*, 47–55, doi:10.1016/0306-4603(82)90024-7.

6. da Luz, F.Q.; Hay, P.; Gibson, A.A.; Touyz, S.W.; Swinbourne, J.M.; Roekenes, J.A.; Sainsbury, A. Does Severe Dietary Energy Restriction Increase Binge Eating in Overweight or Obese Individuals? A Systematic Review. **2015**.
